# Tetramethylpyrazine exerts a protective effect against injury from acute myocardial ischemia by regulating the PI3K/Akt/GSK-3β signaling pathway

**DOI:** 10.1186/s11658-019-0141-5

**Published:** 2019-02-26

**Authors:** Qing Yang, Dan Dan Huang, Da Guang Li, Bo Chen, Ling Min Zhang, Cui Ling Yuan, Hong Hong Huang

**Affiliations:** 1 Blood Transfusion Department, First Hospital of Jilin University, Changchun, Jilin China; 2 Preclinical School of North Sichuan Medical College, Nanchong, Sichuan China; 3 Faculty of Chinese Medical Science, Guangxi University of Chinese Medicine, No. 13 Wuhe Road, Qingxiu District, Nanning, 530222 Guangxi China

**Keywords:** Tetramethylpyrazine, Myocardial ischemia, PI3K, Akt, GSK-3β

## Abstract

**Objective:**

We investigated the protective effect of tetramethylpyrazine (TMP) on injury related to acute myocardial ischemia (AMI) induced by isoproterenol (ISO).

**Materials and methods:**

Rats were randomly assigned to five groups: control, ISO, ISO + propranolol (10 mg/kg), ISO + TMP (10 mg/kg) and ISO + TMP (20 mg/kg). The rats in the three ISO + groups were pretreated with propranolol or TMP, while the rats in the control and ISO groups were pretreated with an equal volume of saline. Afterwards, the rats in the four administration groups were subcutaneously injected with ISO for two consecutive days. The levels of creatine kinase (CK), lactate dehydrogenase (LDH), superoxide dismutase (SOD), malondialdehyde (MDA), tumor necrosis factor-α (TNF-α), interleukin-6 (IL-6) and IL-1β in the serum were measured using ELISA. The expressions of B-cell lymphoma-associated X-2 (Bax-2), B-cell lymphoma-2 (Bcl-2), phosphoinositide-3-kinase (PI3K), protein kinase B (Akt), glycogen synthase kinase 3β (GSK-3β), MDA5 and SOD1 were determined using western blotting assay. The phosphorylation of PI3K, Akt and GSK-3β were also determined using western blotting assay. The left ventricles of the rats were extracted and stained using hematoxylin and eosin (H&E). The ST segment was recorded using electrocardiograms (ECGs).

**Results:**

Administration of TMP (10, 20 mg/kg) reduced the levels of MDA and CK and the activities of SOD and LDH in the serum. Pretreatment with TMP significantly reduced the levels of pro-inflammatory cytokines, including IL-1β, IL-6 and TNF-α. Treatment with TMP also improved the histopathological alteration and decreased the ST elevation. Furthermore, TMP ameliorated the expressions of Cu, SOD1, MDA5, Bax-2, Bcl-2, p-PI3K, p-Akt and p-GSK-3β in ISO-induced rats.

**Conclusions:**

Tetramethylpyrazine protected against injury due to AMI by regulating the PI3K/Akt /GSK-3β signaling pathway.

## Introduction

The leading cause of morbidity in the Western world is acute myocardial ischemia (AMI), which is caused by an imbalance between the blood supply to the heart and the demand of the myocardium [1, 2]. The primary features of AMI are hypoxia, cell ischemia and inflammation. Obstruction of the blood flow to the heart contributes to the ischemia of myocardial cells, which may contribute to the apoptotic process [3–5]. Despite significant research and clinical advances, there have been no fundamental breakthroughs in drug treatment [6].

Isoproterenol (ISO), a β-adrenergic agonist, is known to induce AMI due to autoxidation-related free radical production [7]. ISO-induced AMI increases the levels of cardiac enzymes and oxidative stress, and results in abnormal electrocardiograph and cardiac functions [8].

Although the pathogenesis of AMI is far from clear, the anatomical changes and biochemical markers have been well characterized. Overproduction of reactive oxygen species (ROS) and activation of inflammatory cascades are the major causative factors of cardiomyocyte abnormalities [9, 10]. Activation of the pro-survival kinase-signaling cascade phosphatidylinositol 3-kinase/protein kinase B (PI3K/Akt) promotes cell survival and recruits the anti-apoptotic pathway during reperfusion [11, 12].

Experimental studies have indicated that intervention and ischemia preconditioning using some pharmacological agents can recruit the PI3K/Akt pathway and confer powerful cardioprotection [13]. It has been proposed that pharmacological targeting of the Akt pathway may potentially diminish ischemia reperfusion (IR) injury [14].

Glycogen synthase kinase-3β (GSK-3β) belongs to a family of conserved serine/threonine kinases. Its activity is regulated by the PI3K/Akt, extracellular signal-regulated kinase (ERK1/2) and Wnt/wingless signaling pathways, among others [15]. GSK-3β phosphorylation at Ser9 causes its N-terminal protein tail to act as a pre-phosphorylated substrate, leading to GSK-3β inactivation [16]. The selective inhibition of GSK-3β has been shown to exert cardioprotective effects by maintaining mitochondrial function during ischemia/reperfusion injury [17].

**Fig. 1:**
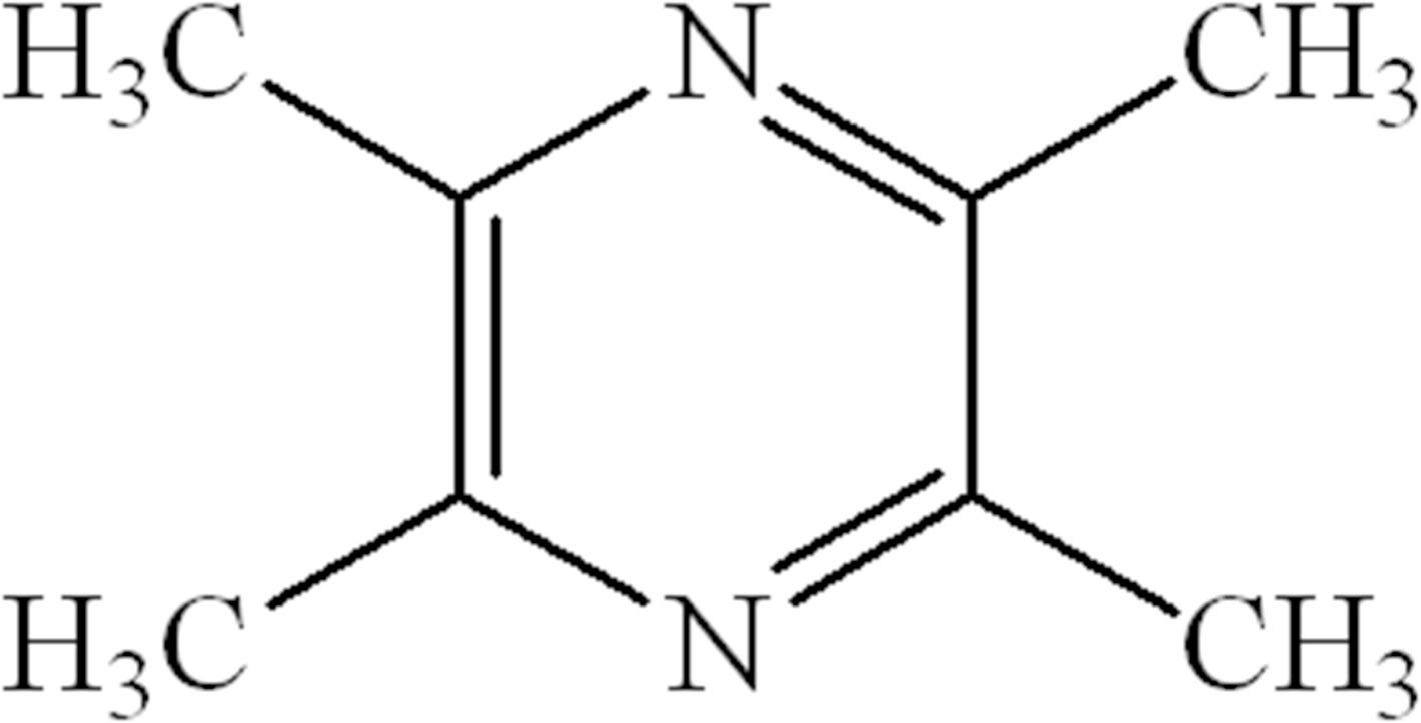
Molecular formula of tetramethylpyrazine (TMP)

Tetramethylpyrazine (TMP) is an alkaloid found in the roots of *Ligusticum chuanxiong* Hort (Fig. 1) [18]. It has cardioprotective effects against myocardial IR injury: it limits infarct size and reduces apoptosis [19]. In this study, we further investigated the cardioprotective effect of TMP and assessed whether the PI3K/Akt/GSK-3β signal pathway was involved in the cardioprotective effect of TMP.

## Materials and methods

### Materials

This study was performed in accordance with the National Institutes of Health Guidelines for the Use of Laboratory Animals. Male Sprague-Dawley (SD) rats (200–220 g) were provided by Shanghai Slac Laboratory Animal Co. Ltd. All animals were allowed free access to food and water, and were maintained at 22–24 °C under a 12 h:12 h light–dark cycle.

Tetramethylpyrazine (TMP; Fig. 1) and isoproterenol (ISO) were obtained from National Institutes for Food and Drug Control in Beijing. Tumor necrosis factor-α (TNF-α), interleukin-6 (IL-6) and IL-1β ELISA kits, creatine kinase (CK), lactate dehydrogenase (LDH), and ELISA kits for the detections of malondialdehyde (MDA) and total superoxide dismutase (T-SOD) were produced by Jiancheng Bioengineering Institute.

### Experimental protocol

Rats were randomly assigned to the control group and four administration groups: ISO, ISO + propranolol (10 mg/kg), ISO + TMP (10 mg/kg), and ISO + TMP (20 mg/kg). There were 10 rats in each group. The rats in the three ISO + groups were pretreated with propranolol or TMP, while the rats in the control and ISO groups were treated with equal volumes of normal saline. Afterwards, the rats in all four administration groups were subcutaneously injected with ISO (85 mg/kg) for two consecutive days [20]. Blood (3 ml) was collected from the abdominal aorta for serum enzyme assays. After treatment, three hearts from each group were weighed and applied for the western blotting assay. Three hearts from each group were rinsed in ice-cold isotonic saline, blotted with filter paper, and homogenized in 0.05 M ice-cold phosphate buffer (pH 7.4) for biochemical assays.

### Evaluation of ST segment elevation

Electrocardiograms (ECGs) recorded ST segment elevation 24 h after the final injection of ISO or other drugs. ECGs were recorded under 3% chloral hydrate anesthesia using needle electrodes and a BL-420S Biological Function Experiment System purchased from Chengdu Thaimeng Technology Co. Ltd.

### Calculation of the heart weight index

The heart tissues were weighed after blotting with filter paper. The heart weight index (HWI) was calculated as heart weight (HW)/bodyweight (BW).

### Determinations of CK, LDH, SOD, MDA, TNF-α, IL-6 and IL-1β in serum

Blood samples were collected from the carotid artery and centrifuged at 3500 rpm for 15 min. Then the supernatants were obtained and preserved at − 80 °C for serum enzyme assays and cytokine analyses. IL-6, IL-1β and TNF-α were analyzed using ELISA kits. The levels of CK, LDH, SOD and MDA were measured using a rate assay. All measurements were performed according to the kit manufacturers’ instructions.

### Histological examination of the myocardium

Immediately after removal, the hearts were fixed in 10% formalin solution. The heart tissue was processed for sectioning and staining using standard histological methods. Sections from the left ventricle were stained using hematoxylin and eosin (H&E).

### Western blotting

Total proteins were extracted from myocardial tissues using ice-cold RIPA lysis buffer. Dissolved proteins were collected and the debris was removed via centrifugation at 12,000 rpm for 5 min at 4 °C. The concentration of total protein was determined using bicinchoninic acid protein assay reagent. Equal amounts of isolated protein were loaded on SDS-polyacrylamide gel electrophoresis (SDS-PAGE) and transferred onto the polyvinylidene difluoride (PVDF) membrane. The membranes were incubated at 4 °C overnight with 5% skim milk in Tris buffer saline for blocking. After the membranes were blocked, they were incubated with monoclonal antibodies against Bax-2, Bcl-2, PI3K, Akt, GSK-3β, p-PI3K, p-Akt, p-GSK-3β, MDA5, SOD1 and GAPDH (all from Cell Signaling Technology). After washing with TBST three times, they were incubated with horseradish peroxidase-conjugated IgGs (1:10000; Bioworld Biotechnology) for 1 h at room temperature. Target proteins were detected using the ECL system (Millipore) and visualized with the ChemiDoc XRS system (Bio-Rad).

### Statistical analysis

Each experiment was repeated at least three times. Data are shown as the means ± standard deviation, and were analyzed using SPSS 18.0. Statistical comparisons between groups were analyzed using two-tailed Student’s t test or one-way ANOVA. *p* < 0.05 is considered to indicate a statistically significant difference.

## Results

### TMP reduces ST segment elevation

**Fig. 2:**
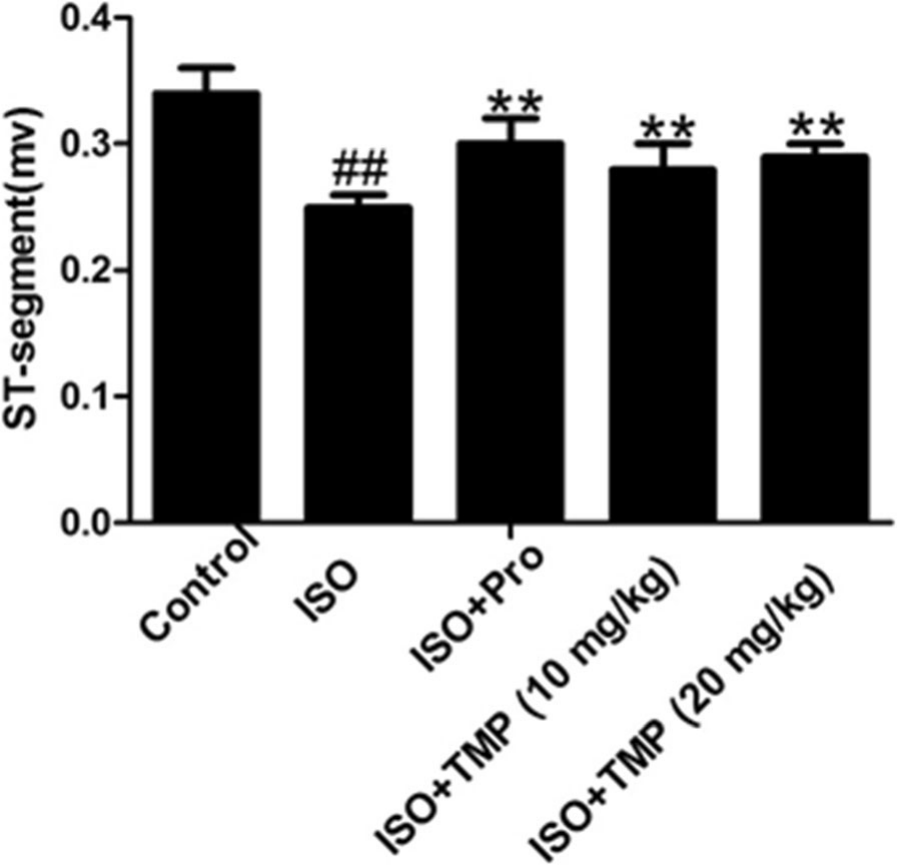
Statistical analysis of tetramethylpyrazine (TMP) on ST segment elevation. Values are expressed as means ± SD. ^##^*p* < 0.01 compared to control, **p < 0.01 compared to ISO group. Control: rats pretreated with saline solution. ISO: rats pretreated with saline solution and treated with isoproterenol (ISO). ISO + Pro: rats pretreated with propranolol (Pro) and treated with ISO. ISO + TMP: rats pretreated with indicated dosage of TMP and treated with ISO

In our study, the ST segment was dramatically elevated in the ISO-stimulated rats. These results suggest that the myocardial ischemia damage model was successfully established. However, the ST segment was obviously decreased in the groups treated with TMP or propranolol compared with those in the ISO group (Fig. 2).

### TMP decreases the levels of TNF-α, IL-6 and IL-1β

**Fig. 3:**
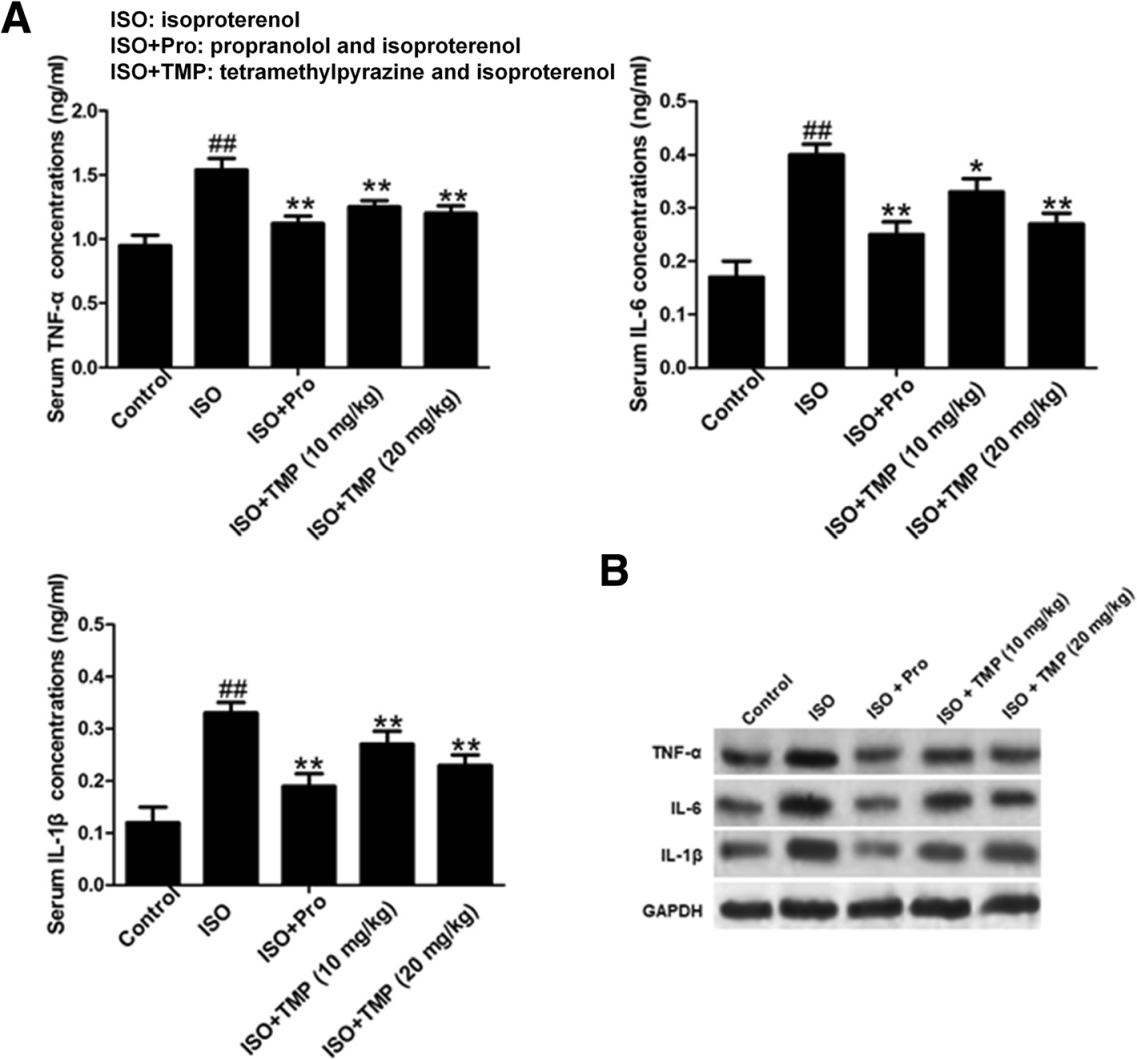
The effects of tetramethylpyrazine (TMP) on the levels of pro-inflammatory cytokines IL-1β, IL-6 and TNF-α in serum were detected using ELISA assays. Values are expressed as means ± SD. ^##^*p* < 0.01compared to control group; *p < 0.05, **p < 0.01 compared to ISO group. Control: rats pretreated with saline solution. ISO: rats pretreated with saline solution and treated with isoproterenol (ISO). ISO + Pro: rats pretreated with propranolol (Pro) and treated with ISO. ISO + TMP: rats pretreated with indicated dosage of TMP and treated with ISO

We also found that the levels of the pro-inflammatory cytokines TNF-α, IL-6 and IL-1β in the serum were significantly increased in ISO rats compared with the control group. However, pretreatment with TMP decreased the levels of TNF-α, IL-6 and IL-1β relative to the levels for untreated rats in a dose-dependent manner (Fig. 3).

### TMP decreases CK and LDH serum levels

**Fig. 4:**
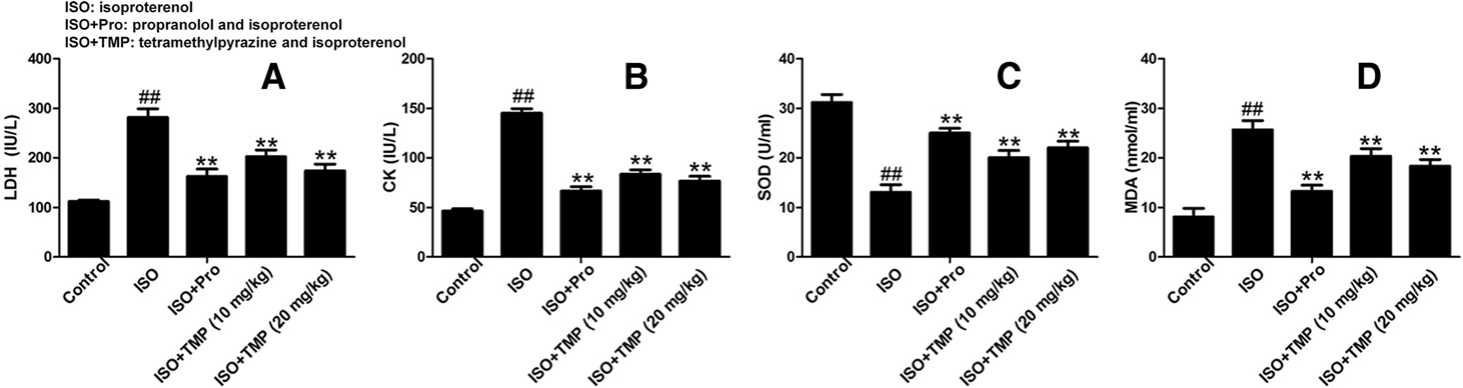
The effects of tetramethylpyrazine (TMP) on the levels of creatine kinase (CK), lactate dehydrogenase (LDH), superoxide dismutase (SOD) and malondialdehyde (MDA) in the serum. **a** The effects of TMP on the levels of LDH in the serum were analyzed using ELISA. **b** The effects of TMP on the levels of CK in the serum were analyzed using ELISA. **c** The effects of TMP on the levels of SOD in the serum were analyzed using ELISA. **d** The effects of TMP on the levels of MDA in the serum were analyzed using ELISA. Control: rats pretreated with saline solution. ISO: rats pretreated with saline solution and treated with isoproterenol (ISO). ISO+Pro: rats pretreated with propranolol (Pro) and treated with ISO. ISO+TMP: rats pretreated with indicated dosage of TMP and treated with ISO.Values are expressed as means ± SD. ##*p* < 0.01 compared to control group; *p < 0.05, **p < 0.01 compared to ISO group

The serum levels of CK and LDH, which are marker enzymes of myocardial injury, were also examined. The levels of CK and LDH increased in the ISO group in comparison with those in the control group (Fig. 4a and b). Propranolol could decrease the contents of CK and LDH. Pretreatment with TMP decreased CK and LDH levels compared with the levels for rats in the ISO group in a dose-dependent manner.

### TMP decreases SOD and MDA serum levels

To determine whether TMP attenuated the oxidation index, the levels of SOD and MDA in the serum were measured. Compared with the control group, SOD levels in the ISO group were significantly downregulated by TMP (Fig. 4c). However, pretreatment with TMP markedly increased the level of SOD (Fig. 4c) and reduced the MDA level (Fig. 4d) in a dose-dependent manner. These data suggest that TMP significantly enhanced the activities of the enzymatic antioxidant defense system.

### TMP decreases heart weight indices

**Fig. 5:**
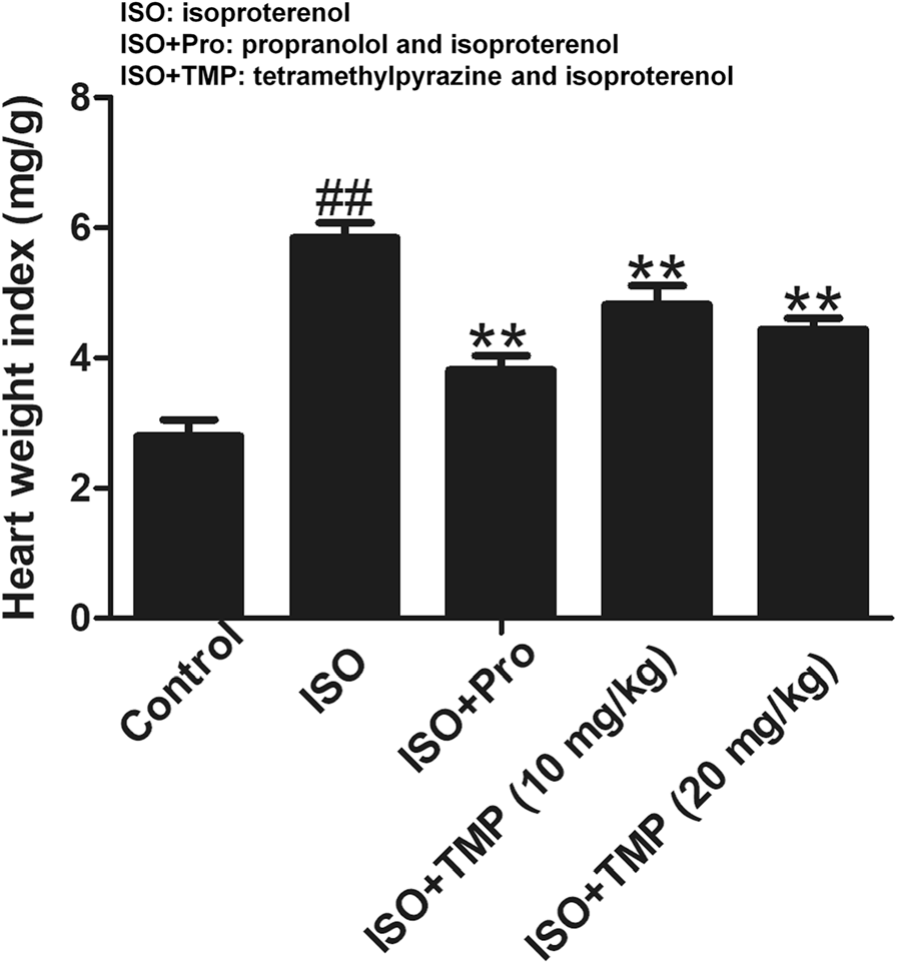
Effects of tetramethylpyrazine (TMP) on heart weight indices. Values are expressed as means ± SD. ^##^*p* < 0.01 compared to control group; *p < 0.05, **p < 0.01 compared to ISO group. Control: rats pretreated with saline solution. ISO: rats pretreated with saline solution and treated with isoproterenol (ISO). ISO + Pro: rats pretreated with propranolol (Pro) and treated with ISO. ISO + TMP: rats pretreated with indicated dosage of TMP and treated with ISO

Heart weight indices (HWIs) were measured to assess injury due to acute myocardial ischemia (AMI). The HWIs for the ISO group rats were higher than those for the control group rats. Pretreatment with TMP decreased the HWIs compared with those for the ISO group rats (Fig. 5).

### TMP attenuates the histopathological condition of myocardial tissue

**Fig. 6:**
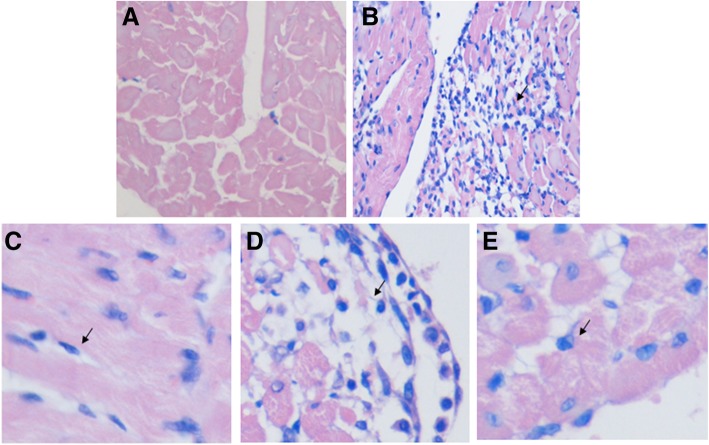
Effects of TMP on myocardial histology. **a** Control group. **b** ISO group (rats pretreated with saline solution and treated with ISO). **c** ISO + Pro group (rats pretreated with propranolol (Pro) and treated with ISO). **d** ISO + TMP (10 mg/kg) group (rats pretreated with 10 mg/kg TMP and treated with ISO). **e** ISO + TMP (20 mg/kg) group (rats pretreated with 20 mg/kg TMP and treated with ISO)

Hematoxylin and eosin (H&E) staining was carried out to evaluate the protective role of TMP. Light microscopy of tissue sections from control rat myocardia showed a normal myofibrillar structure with striations, a branched appearance and continuity with adjacent myofibrils. The pathology assessment of the myocardia of ISO rats revealed obvious myocardial cell swelling, degeneration, transverse striation loss and inflammatory cell infiltration. Tissues from rats pretreated with TMP showed normal, well preserved cardiac muscle cell histology. Tissue sections from the propranolol group rats revealed approximately normal myofibrillar structure with clear transverse striations and the presence of a few inflammatory cells (Fig. 6). The results indicate that TMP could attenuate the histopathological condition in myocardial tissue.

### TMP decreases the levels of MDA5 and increases the levels of SOD1

**Fig. 7:**
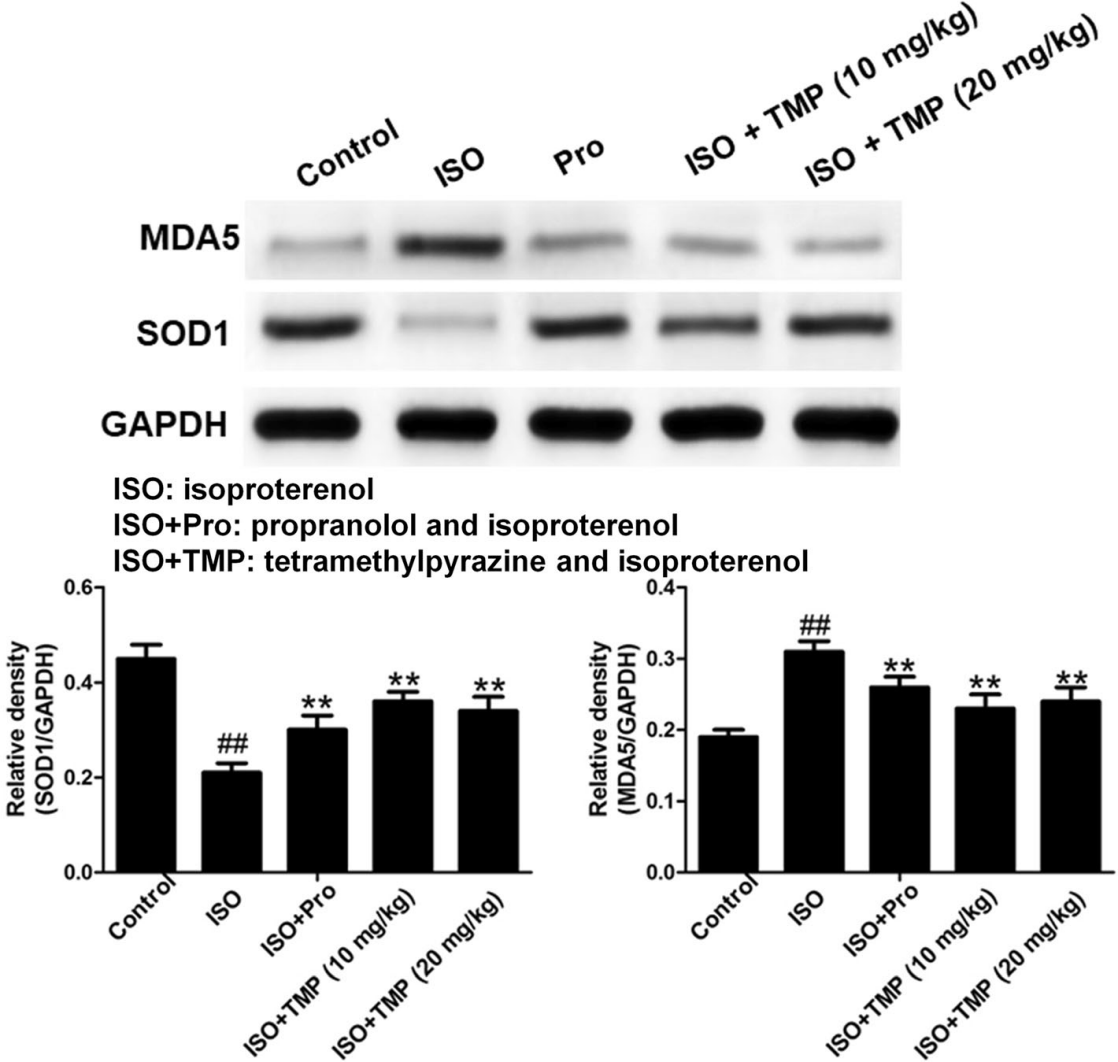
The effects of tetramethylpyrazine (TMP) on the expression of SOD1 (A) and MDA5 (B) were measured using a western blotting assay. Values are expressed as means ± SD. ^#^*p* < 0.05, ^##^*p* < 0.01 compared to control group. *p < 0.05, **p < 0.01compared to ISO group. Control: rats pretreated with saline solution. ISO: rats pretreated with saline solution and treated with isoproterenol (ISO). ISO + Pro and Pro: rats pretreated with propranolol (Pro) and treated with ISO. ISO + TMP: rats pretreated with indicated dosage of TMP and treated with ISO

ISO also changed the protein expressions of SOD1 and MDA5 in heart tissue. Compared with the control group, the protein levels of MDA5 in the ISO group were significantly higher (Fig. 7). In the TMP-pretreated group, the protein levels of MDA5 were significantly lower. Compared with the control group, the protein levels of SOD1 in the ISO group were significantly lower. In the TMP-pretreated group, the protein levels of SOD1 were significantly higher than in the ISO group. The results confirmed the anti-oxidative effect of TMP on ISO-induced AMI.

### TMP decreases Bax-2 levels and increases PI3K, Akt, GSK-3β and Bcl-2 levels

**Fig. 8:**
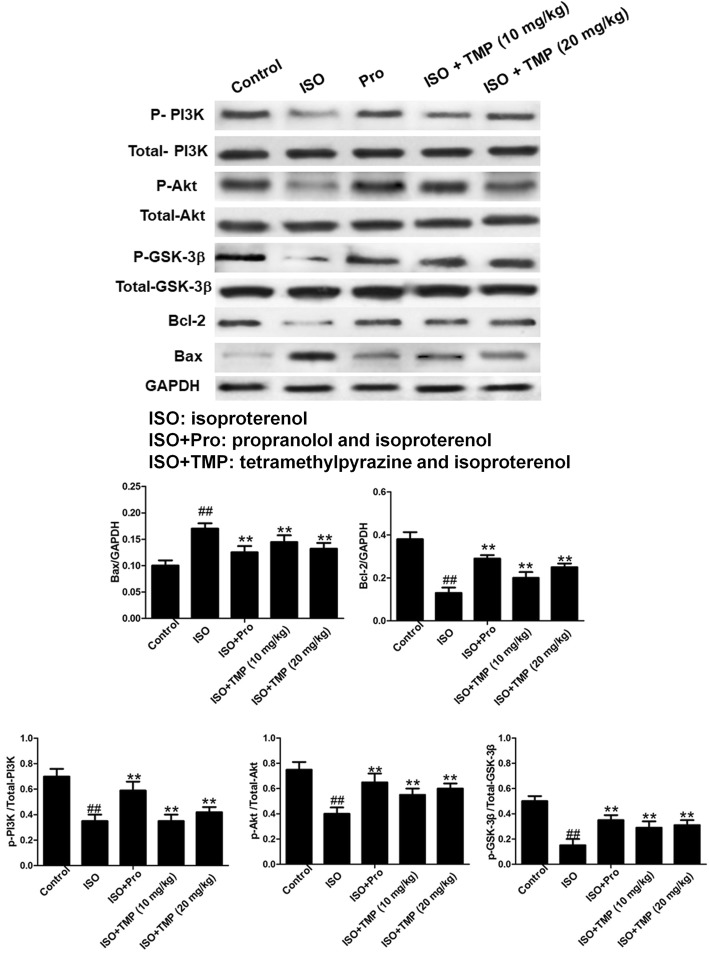
The effects of tetramethylpyrazine (TMP) on the PI3K/Akt/GSK-3β pathway. The phosphorylation of PI3K, Akt and GSK-3 were detected using a western blotting assay. The expression of Bax-2, Bcl-2, PI3K, Akt, GSK-3β, MDA5 and SOD1 were detected using a western blotting assay. Values are expressed as means ± SD. ^#^*p* < 0.05, ^##^*p* < 0.01 compared to control group. *p < 0.05, **p < 0.01compared to ISO group. Control: rats pretreated with saline solution. ISO: rats pretreated with saline solution and treated with isoproterenol (ISO). ISO + Pro and Pro: rats pretreated with propranolol (Pro) and treated with ISO. ISO + TMP: rats pretreated with indicated dosage of TMP and treated with ISO

To further investigate the potential protective mechanism, the impact of TMP on PI3K/Akt/GSK-3β signaling was determined using western blotting assay. The expressions of Bcl-2, p-PI3K, p-Akt and p-GSK-3β in the myocardia of ISO rats were significantly downregulated in contrast with those in the control group (Fig. 8). In the TMP-pretreated group, the protein levels of Bcl-2, p-PI3K, p-Akt and p-GSK-3β significantly increased in a dose-dependent manner compared to the levels for the model group.

## Discussion

During acute myocardial ischemia (AMI), ST segment elevation is closely associated with myocardial blood flow, oxygen tension and the contraction rate. Myocardial ischemia induced by coronary artery ligation in rats shows very similar characteristics and can be used as a model of AMI [21]. ST segment elevation is the most straightforward way to identify myocardial ischemia, as shown in the records of electrocardiographs (ECGs) in this study. In our tetramethylpyrazine (TMP) groups, the ST segment was effectively attenuated, suggesting that TMP has a positive influence on cardiac electrical activity.

Creatine kinase (CK) and lactate dehydrogenase (LDH) are identified as essential serum biomarkers for the diagnosis of AMI [22]. The abnormal myocardial energy metabolism seen during AMI elevates the CK level in the serum [23]. Myocardial necrosis enhances the membrane permeability that leads to excessive release of LDH [24]. Our results show that TMP pretreatment potently reduced the levels of LDH and CK after MI was induced. These findings aligned with the histological evaluation of myocardial tissues.

Enhanced levels of inflammatory markers are related to ischemia [25, 26]. Pro-inflammatory cytokines, such as IL-6, IL-1β and TNF-α, are small secreted proteins that mediate and regulate inflammation [27]. In our study, TMP decreased the levels of pro-inflammatory cytokines, suggesting that the cardioprotective effects were related to anti-inflammatory properties.

Superoxide dismutase (SOD) is an important antioxidant enzyme that reflects the capacity of the cell to scavenge free radicals [28]. Under physiological conditions, low levels of reactive oxygen species play important roles in signal transduction and metabolic pathways. However, under pathological conditions, excessive reactive oxygen species result in an imbalance of the antioxidant system. It was proposed that malondialdehyde (MDA) caused injury to the mitochondria and lysosome [29]. SOD1 scavenges superoxide anion to prevent oxygen stress injury and facilitates the generation of SOD [30]. Similarly, the antioxidant protein MDA5 promotes the production of MDA. SOD1 gains importance in the development of heart failure. The alterations in SOD, SOD1, MDA and MDA5 levels support our hypothesis that the cardioprotective effects of TMP in ISO-induced AMI are related to anti-oxidative properties [31].

As part of the intrinsic apoptosis pathway, the Bcl-2 protein family is an important regulator during cardiomyocyte apoptosis [32]. The anti-apoptotic protein, Bcl-2, and survivin have been shown to block the release of cytochrome c from the mitochondria, inhibit caspase activity, and decrease cell apoptosis [33]. Therefore, the balance in apoptotic signaling is influenced by the Bcl-2-to-Bax ratio. Western blotting revealed that TMP increased Bcl-2 and decreased Bax-2 levels, suggesting that TMP-mediated cardioprotection against acute myocardial ischemia injury may occur partially via modulation of Bcl-2 and Bax expression.

Activation of the pro-survival kinase-signaling cascade PI3K/Akt at the time of reperfusion promotes cell survival and recruits the anti-apoptotic pathway during reperfusion [34]. Experimental studies have indicated that the intervention and ischemia preconditioning of some pharmacological agents can recruit the PI3K/Akt pathway and confer powerful cardioprotection [35]. The serine/threonine survival kinase GSK-3β is a point of downstream convergence for PI3K/Akt and Wnt signaling. It is known that ischemic pre- and post-conditioning activate PI3K/Akt and promote the phosphorylation of target molecules, such as GSK-3β, that are downstream of insulin receptor substrates [36]. As depicted in Fig. 7, the expressions of Bax-2 in the myocardia of the ISO rats were significantly upregulated in contrast with those in control group. Compared with the control group, the protein levels of PI3K, Akt, GSK-3β and Bcl-2 in ISO group were significantly lower. In groups with TMP pretreatment, the protein levels of PI3K, Akt, GSK-3β and Bcl-2 significantly increased in a dose-dependent manner compared to the ISO group.

## Conclusion

ISO-induced AMI could be attenuated by TMP through regulation of the inflammatory condition and oxidative stress. Our study also demonstrated that TMP exerted cardioprotective effects via regulation of the PI3K/Akt/GSK-3β pathway.

## Abbreviations

AMI: Acute myocardial ischemia
CGs: Electrocardiograms
CK: Creatine kinase
H&E: Hematoxylin and eosin
IL-1β: Interleukin-1 beta
IL-6: Interleukin-6
ISO: Isoproterenol
LDH: Lactate dehydrogenase
MDA: Malondialdehyde
SOD: Superoxide dismutase
TMP: Tetramethylpyrazine
TNF-α: Tumor necrosis factor-alpha
